# Identification of telocytes in dystrophic mice testis

**DOI:** 10.31744/einstein_journal/2021AI5737

**Published:** 2021-01-22

**Authors:** Vilessa Lilian de Araújo Gomes, Janine Karla França da Silva Braz, Gabriel Moura Martins, Naianne Kelly Clebis, Moacir Franco de Oliveira, Danielle Barbosa Morais, Carlos Eduardo Bezerra de Moura

**Affiliations:** 1 Universidade Federal de Campina Grande Campina GrandePB Brazil Universidade Federal de Campina Grande , Campina Grande , PB , Brazil .; 2 Universidade Federal do Rio Grande do Norte NatalRN Brazil Universidade Federal do Rio Grande do Norte , Natal , RN , Brazil .; 3 Universidade Federal Rural do Semi-Árido MossoróRN Brazil Universidade Federal Rural do Semi-Árido , Mossoró , RN , Brazil .

**Figure 1:**
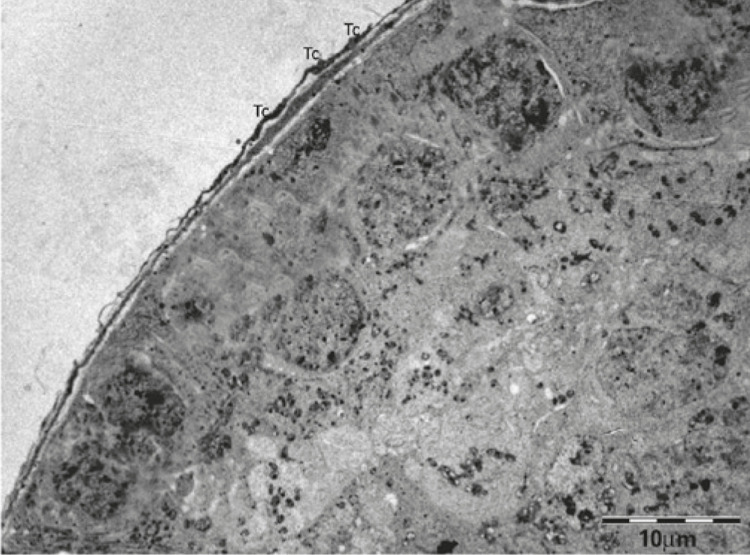
Telocytes surrounding seminiferous tubule in testis of dystrophic mouse

**Figure 2:**
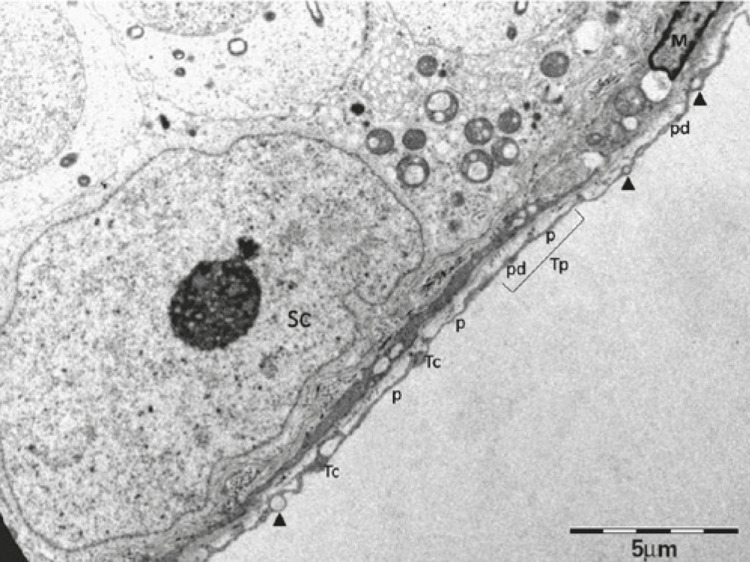
Telocytes in peritubular cells of testis of dystrophic mouse

**Figure 3:**
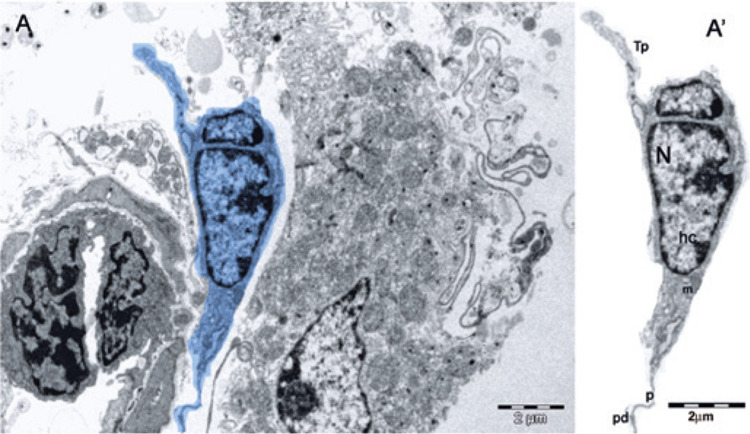
Telocytes in intertubular spaces of testis of dystrophic mouse. (A) digitally colored transmission electron microscope of telocyte (blue); (A’) section of telocyte with oval nucleus and peripheral heterochromatin

Telocyte (Tc) is a new type of classic interstitial cells, described by Popescu et al., ^( 1 )^ in the human pancreas. Telocytes are found in several organs, including the human testis. ^( 2 )^ They are long, thin cells ( Figure 1 ), with small cell bodies, a nucleus containing heterochromatin and presence of mitochondria in the periphery, in addition to a scarcely evident nucleolus. ^( 3 - 5 )^ They differ from other interstitial cells by their long moniliform cytoplasmic extensions, called telopodes (Tp) ( Figure 2 ), and are usually identified through their ultrastructure by transmission electron microscopy (TEM). ^( 1 - 5 )^ The long cytoplasmic projections, Tp, comprise thin segments (podomeres) and dilated regions (podoms), containing secretory vesicles and mitochondria ^( 4 , 5 )^ ( Figure 2 ). Telocytes are located in the peritubular region of the testis and contact myoid cells through cell junctions, while also contacting blood vessels and androgen-producing interstitial cells (Leydig cells) through Tp. ^( 2 , 3 , 5 , 6 )^ Therefore, it is believed that Tc establish homo- and heterocellular junctions, vesicle release and paracrine and/or autocrine signaling. They also interact and communicate with Leydig myoid cells and blood vessels through Tp, being responsible for the transport of substances between the interstitium (Figure 3A) and the seminiferous tubule, such as testosterone, which is essential for spermatogenesis. ^( 2 - 5 )^

Tc: telocyte.

Arrow head: vesicles. Tc: telocyte; p: podomer; pd: podom; Tp: telopodes; SC: Sertoli cell; M: single peritubular myoid cell.

Tp: telopodes; N: nucleus; hc: peripheral heterochromatin; m: mitochondria; p: podomer; pd: podom.

Dystrophic intertubular spaces contain Tc measuring, in average, approximately 8µm. ^( 7 )^ On the scale of 2µm, it is possible to identify mitochondria, an irregular and oval nucleus with peripheral heterochromatin, telopodes, podom and podomer (Figure 3A’). ^( 8 )^ In this context, we could demonstrate Tc, for the first time, in 12 testis of mdx mice with Duchenne muscular dystrophy through TEM analyses. Duchenne muscular dystrophy is a degenerative, progressive and disabling genetic disorder linked to the X chromosome, which does not cause infertility. ^( 9 )^ Thus, evidence of Tc in the testis of dystrophic mice implies in further understanding of spermatogenesis, since these cells help transporting testosterone to the seminiferous tubule.
