# Supplementary material for: Identification of telocytes in dystrophic mice testis
Source: Einstein (Sao Paulo). 2021 Jan 22;19:eAI5737. doi: 10.31744/einstein_journal/2021AI5737 (PMC7821952; doi:10.31744/einstein_journal/2021AI5737)
Supplement: Translation

# Translation: Identificação de telócitos em testículos de camundongos distróficos

**Authors:** Vilessa Lilian de Araújo Gomes, Janine Karla França da Silva Braz, Gabriel Moura Martins, Naianne Kelly Clebis, Moacir Franco de Oliveira, Danielle Barbosa Morais, Carlos Eduardo Bezerra de Moura

**Affiliations:** 1 Brazil Universidade Federal de Campina Grande, Campina Grande, PB, Brazil .; 2 Brazil Universidade Federal do Rio Grande do Norte, Natal, RN, Brazil .; 3 Brazil Universidade Federal Rural do Semi-Árido, Mossoró, RN, Brazil .

O telócito (Tc) é um novo tipo de célula intersticial clássica, descrita por Popescu et al., ^( 1 )^ no pâncreas humano. Os Tc são encontrados em diversos órgãos, inclusive nos testículos humanos. ^( 2 )^ São células longas e finas ( Figura 1 ), com pequenos corpos celulares, um núcleo com heterocromatina e presença de mitocôndria na periferia, além de um nucléolo pouco evidente. ^( 3 - 5 )^ Eles se diferenciam de outras células intersticiais por suas extensões moniliformes citoplasmáticas longas, chamadas telopódios (Tp) ( Figura 2 ), e são geralmente identificados por meio da ultraestrutura na Microscopia Eletrônica de Transmissão (MET). ^( 1 - 5 )^ As longas projeções citoplasmáticas Tp contêm segmentos afilados (podômeros) e regiões dilatadas ( *podoms* ), com vesículas secretoras e mitocôndria ^( 4 , 5 )^ ( Figura 2 ). Os Tcs estão localizados na região peritubular dos testículos e entram em contato com células mioides por meio das junções celulares, quando também contatam os vasos sanguíneos e as células intersticiais produtoras de androgênio (células de Leydig) por meio de Tp. ^( 2 , 3 , 5 , 6 )^ Portanto, acredita-se que o Tc realiza junções homo- e heterocelulares, liberação de vesículas e sinalização parácrina e/ou autócrina. Também interage e comunica com as células mioides, de Leydig e os vasos sanguíneos pelos Tp, sendo responsáveis pelo transporte de substâncias entre o interstício (Figura 3A) e o túbulo seminífero, como a testosterona, que é essencial para espermatogênese. ^( 2 - 5 )^

**Figura 1:** Telócitos ao redor de túbulo seminífero em testículo de camundongo distrófico

Tc: telócito.

**Figura 2:** Telócitos em células peritubulares de testículo de camundongo distrófico

Vesículas (ponta da seta). Tc: telócito; p: podômero; pd: podom; Tp: telopódios; SC: célula de Sertoli; M: célula mioide peritubular única.

**Figura 3:** Telócitos em espaços intertubulares de testículo de camundongo distrófico. (A) microscopia eletrônica de transmissão com coloração digital de telócito (azul); (A’) corte de telócito com núcleo oval e heterocromatina periférica

Tp: telopódios; N: núcleo; hc: heterocromatina periférica; m: mitocôndria; p: podômero; pd: podom.

Os espaços intertubulares distróficos contêm Tc, que medem, em média, aproximadamente 8µm. ^( 7 )^ Na escala de 2µm, é possível identificar a mitocôndria, o núcleo irregular e oval com heterocromatina periférica, o Tp, o podom e o podômero (Figura 3A’). ^( 8 )^ Nesse contexto, pudemos pela primeira vez demonstrar Tc em 12 testículos de camundongos mdx com distrofia muscular de Duchenne, por análises de MET. A distrofia muscular de Duchenne é um doença genética ligada ao cromossomo X, degenerativa, progressiva e incapacitante, que não causa infertilidade. ^( 9 )^ Assim, evidência de Tc nos testículos de camundongos distróficos leva à maior compreensão da espermatogênese, pois essas células auxiliam no transporte de testosterona para o túbulo seminífero.
